# Cystatin C as a potential predictor of osteoprotegerin levels in healthy men, a cross-sectional, observational study

**DOI:** 10.1186/s12891-015-0684-1

**Published:** 2015-08-28

**Authors:** Eva Kulcsar-Jakab, Zsofia Petho, Zoltan Pap, Edit Kalina, Roza Foldesi, Adam Balogh, Peter Antal-Szalmas, Harjit Pal Bhattoa

**Affiliations:** Department of Laboratory Medicine, Faculty of Medicine, University of Debrecen, Debrecen, Hungary; Department of Rheumatology, Faculty of Medicine, University of Debrecen, Debrecen, Hungary; Department of Traumatology and Hand Surgery, Faculty of Medicine, University of Debrecen, Debrecen, Hungary; Regional Osteoporosis Center, Department of Obstetrics and Gynecology, Faculty of Medicine, University of Debrecen, Debrecen, Hungary

## Abstract

**Background:**

The aim of the present study is to evaluate serum osteoprotegerin (OPG) and soluble receptor activator of nuclear factor κB ligand (sRANKL) levels in a randomly selected male cohort over 50 years of age and its association with cystatin C, a cysteine proteinase inhibitor that decreases formation of osteoclasts by interfering at a late stage of pre-osteoclast differentiation, apart from being a marker of renal function independent of gender, muscle mass and age; in addition to known predictors such as age, sex hormones, vitamin D, bone mineral density (BMD) and biochemical markers of bone turnover.

**Methods:**

We determined serum OPG and sRANKL levels and examined its relationship with cystatin C, age, osteocalcin, C-terminal telopeptides of type-I collagen, procollagen type 1 amino-terminal propeptide, 25-hydroxyvitamin D, parathyroid hormone, total 17β-estradiol (E2), total testosterone and L1–L4 (LS) and femur neck (FN) BMD data available from 194 (age, range: 51–81 years) randomly selected ambulatory men belonging to the HunMen cohort.

**Results:**

OPG correlated significantly with age (Spearman’s rho (r) = 0.359, *p* < 0.001), cystatin C (*r* = 0.298, *p* < 0.001), E2 (*r* = 0.160, *p* = 0.028) and free testosterone index (FTI) (*r* = −0.230, *p* = 0.001). Compared to the middle-aged (age: ≤ 59 years, *n* = 98), older men (age > 59 years, *n* = 96) had significantly higher serum OPG (4.6 pmol/L vs. 5.4 pmol/L; *p* < 0.001), and lower sRANKL (0.226 pmol/L vs. 0.167 pmol/L; *p* = 0.048) levels. The older men showed a significant correlation between serum OPG levels and cystatin C (Spearman’s rho = 0.322, *p* = 0.002), and E2 (Spearman’s rho = 0.211, *p* = 0.043). Including cystatin C and E2 in a regression model showed that cystatin C (standard regression coefficient (β) = 0.345; *p* = 0.002) was the only significant predictor of serum OPG levels in the older men.

**Conclusions:**

The results of this study demonstrated that in addition to age (which was the stronger predictor), other modifiable factors such as cystatin C, FTI and E2 were also significant predictors of OPG, and that the association between cystatin C and OPG was more evident with increased age (older age group). As such, cystatin C is a significant predictor of OPG independently of age, FTI and E2.

**Electronic supplementary material:**

The online version of this article (doi:10.1186/s12891-015-0684-1) contains supplementary material, which is available to authorized users.

## Background

Osteoprotegerin (OPG) and receptor activator of nuclear factor-κB ligand (RANKL), two members of the tumor necrosis factor receptor super family, are essential in the regulation of bone resorption [1, 2]. OPG is a soluble decoy receptor, secreted by osteoblasts and other cell types, and competitively binds RANKL. RANKL is expressed primarily by osteoblasts and its receptor RANK is expressed in preosteoclasts and other cells of this lineage. When RANKL binds to RANK, osteoclastogenesis and bone resorption are induced. Thus, bone resorption may be prevented by OPG binding to RANKL [3]. In addition to being a key regulator of osteoclastogenesis, the OPG/RANKL/RANK system is reported as being a significant mediator between factors such as estradiol, testosterone, parathyroid hormone, vitamin D, and bone turnover [3–6].

Data on OPG and RANKL levels in healthy men is accumulating [7–19]. Nonetheless, based on published data, it may well be generalized that the association between OPG/RANKL and its predictors is lacking consensus. Furthermore, the association of cystatin C with OPG and RANKL has only been limitedly investigated in the healthy, where no significant correlation was found [14]. Nonetheless, in-vitro studies have implied that cystatin C is a cysteine proteinase inhibitor that decreases osteoclastogenesis by interfering at a late stage of pre-osteoclast differentiation [20, 21]. Additionally, cystatin C possesses the advantage of being independent of gender, muscle mass and age; and consequently may qualify better than creatinine as a surrogate marker of renal function in investigating the role of decreased protein clearance as a potential cause of age-related OPG elevation [22]. The aim of the present study is to evaluate serum OPG and RANKL levels in a randomly selected male cohort over 50 years of age and its association with cystatin C, age, sex hormones, vitamin D, bone mineral density (BMD) and biochemical markers of bone turnover.

## Methods

### Subjects

The fasting serum samples (*n* = 194) for OPG and soluble RANKL (sRANKL) determinations belonged to participants in the HunMen cross-sectional, observational study [23]. Briefly the HunMen study was a local initiative to evaluate the bone health of randomly selected healthy men who confirmed to the inclusion and/or did not confirm to the exclusion criteria. Recruitment was between September 2009 and September 2010. Inclusion criteria were: over 50 years of age, male, ambulatory, community dwelling and generally regarded as healthy. Exclusion criteria were: known prevalent metabolic bone disease, liver or renal disease and use of medication influencing bone metabolism (excluding calcium and vitamin D supplementation). The study was approved by the ethics review board of the University of Debrecen, Hungary in compliance with the Declaration of Helsinki, and all subjects gave written informed consent.

### Study procedures

Dual energy X-ray absorptiometry examination was performed using the LUNAR Prodigy (GE-Lunar Corp., Madison, Wisc., USA) densitometer. BMD was measured at L1–L4 lumbar spine (LS) and femur neck (FN). The coefficient of variation (CV) of the technique at our institute was 0.8 % using the anatomical spine phantom measured daily.

Serum total OPG and sRANKL were measured using enzyme immunoassays (Biomedica Gruppe, Biomedica Medizinprodukte GmbH & Co KG, Wein, Austria). The OPG assay, that detects monomeric dimeric and ligand bound OPG, uses a monoclonal mouse anti-OPG antibody as capture antibody and a biotin-labeled goat polyclonal antibody for detection. The sRANKL assay, that detects soluble, uncomplexed human RANKL, uses human recombinant OPG for capture and a biotin-labeled goat polyclonal antibody for detection. Plasma 25-hydroxyvitamin D (25-OH-D) was analyzed by high pressure liquid chromatography (HPLC) using a Jasco HPLC system (Jasco, Tokyo, Japan) and Bio-Rad reagent kit (Bio-Rad Laboratories, Hercules, CA, USA). Serum estradiol (E2), testosterone (T), sex hormone binding globulin (SHBG), parathyroid hormone (PTH), osteocalcin (OC), C-terminal telopeptides of type-I collagen (CTx), procollagen type 1 amino-terminal propeptide (PINP) were measured using electrochemiluminiscence immunoassay (Roche Diagnostics GmbH, Mannheim, Germany). The inter-assay CV was <8 % for OPG (lower detection limit: 0.14 pmol/L, upper detection limit: 30 pmol/L), <6 % for sRANKL (lower detection limit: 0.02 pmol/L, upper detection limit: 2 pmol/L), <3.5 % for 25-OH-D (25-OH-D2 - lower detection limit: 13.1 nmol/L, upper detection limit: 606 nmol/L; 25-OH-D3 - lower detection limit: 16.5 nmol/L, upper detection limit: 624 nmol/L), <7 % for E2 (lower detection limit: 0.018 nmol/L, upper detection limit: 15.78 nmol/L), <9 % for T (lower detection limit: 0.087 nmol/L, upper detection limit: 52 nmol/L), <6 % for SHBG (lower detection limit: 0.350 nmol/L, upper detection limit: 200 nmol/L), <7 % for PTH (lower detection limit: 0.127 pmol/L, upper detection limit: 530 pmol/L), <4 % for OC (lower detection limit: 0.5 μg/L, upper detection limit: 300 μg/L), <7 % for CTx (lower detection limit: 0.010 μg/L, upper detection limit: 6 μg/L) and <6 % for PINP (lower detection limit: 5 μg/L, upper detection limit: 1200 μg/L).

Plasma cystatin C was measured using particle-enhanced nephelometric immunoassay (Siemens Healthcare Diagnostics Products GmbH, Marburg, Germany), its inter-assay CV was <4 % (lower detection limit: 0.05 mg/L, upper detection limit: 7.58 mg/L). Creatinine was measured using the Creatinine Jaffé 2nd generation (compensated) test on the cobas c 111 system (Roche Diagnostics GmbH, Mannheim, Germany). The free estradiol index (FEI) and free testosterone index (FTI) was calculated as the total 17β-estradiol to SHBG ratio and total testosterone to SHBG ratio, respectively. The 4-variable modification of diet in renal disease (MDRD) study equation was used for calculating eGFR [24]. Additionally, eGFR was also calculated using the chronic kidney disease-epidemiology collaboration (CKD-EPI) cystatin C and CKD-EPI creatinine-cystatin C equations [25, 26].

### Statistical analysis

Kolmogorov-Smirnov test was used for the evaluation of the normality of the data. Most parameters were non-normally distributed; therefore analyses were performed by Mann–Whitney *U* test. The Spearman’s rho was calculated for correlation analysis. Associations were tested using linear regression analysis after log transformation of not normal variables. Additionally, to reduce the confounding effect of age, the median age was used to form two subgroups. A value of *p* < 0.05 was considered statistically significant. All analyses were performed with the SPSS Statistics software, version 19.0 (IBM Corps., Armonk, NY, USA).

## Results

In the HunMen study, a total of 229 randomly selected volunteers agreed to participate. volunteers not confirming to the inclusion and/or confirming to the exclusion criteria (*n* = 23) were excluded from the study’s final statistical analysis. The HunMen study was not designed primarily for the aim of this study, and only 194 samples were available for serum OPG and sRANKL determination from a total of 206 participants. The general characteristics of the subjects (*n* = 194) are presented in Table 1.

**Table 1:** Subject characteristics

	All men (*n* = 194)	Middle-aged men (≤59 years of age, *n* = 98)	Older men (>59 years of age, *n* = 96)
Age, years (mean, range)	60.4 (51–81)	55.6 (51–59)*	65.4 (60–81)*
Body mass index, kg/m^2^ (mean, range)	29.2 (17.3–41.7)	28.9 (18–38.5)	29.5 (17.3–41.7)
Osteoprotegerin, pmol/L (mean, range)	5.0 (2.2–10.1)	4.6 (2.2–7.7)**	5.4 (3.0–10.1)**
sRANKL, pmol/L (mean, range) (≥0.020 pmol/L detection limit)	0.197 (0.020–0.875) (*n* = 120)	0.226 (0.026–0.875)*** (*n* = 61)	0.167 (0.020–0.795)*** (*n* = 59)
sRANKL/Osteoprotegerin ratio (mean, range)	0.043 (0.003–0.256) (*n* = 120)	0.053 (0.006–0.256)**** (*n* = 61)	0.033 (0.003–0.176)**** (*n* = 59)
Total 17-β-Estradiol, nmol/L (mean, range)	0.09 (0.02–0.19)	0.09 (0.03–0.19)	0.08 (0.02–0.15)
Total testosterone, nmol/L (mean, range)	12.8 (0.3–41.7)	13.1 (2.7–41.7)	12.4 (0.3–32.1)
Sex hormone binding globulin, nmol/L (mean, range)	40.8 (12.3–196.2)	40.7 (12.7–196.2)	40.9 (12.3–115.5)
Free Estradiol Index (mean, range)	0.0026 (0–0.009)	0.0026 (0.001–0.007)	0.0025 (0–0.009)
Free testosterone Index (mean, range)	0.3398 (0.005–0.743)	0.354 (0.081–0.704)*****	0.325 (0.005–0.743)*****
PTH, pmol/L (mean, range)	4.2 (1–10.2)	4.2 (1–10.2)	4.3 (1.6–8.3)
25-hydroxy-vitamin D, nmol/L (mean, range)	73.1 (11–185)	72.1 (11–137)	74.0 (14–185)
Osteocalcin, μg/L (mean, range)	14.6 (5–35)	15.2 (5–33)	14.0 (6–35)
C-terminal telopeptides of type-I collagen, μg/L (mean, range)	0.22 (0.01–0.77)	0.24 (0.01–0.77)	0.20 (0.04–0.70)
PINP, μg/L (mean, range)	38.3 (10.5–98.6)	39.2 (10.5–98.6)	37.3 (13.1–81.3)
Cystatin C, mg/L (mean, range)	0.697 (0.381–1.150)	0.657 (0.381–1.060)******	0.736 (0.485–1.150)******
L1-L4 bone mineral density, gm/cm^2^ (mean ± SD)	1.172 ± 0.185	1.148 ± 0.167	1.197 ± 0.199
Femur neck bone mineral density, gm/cm^2^ (mean ± SD)	0.970 ± 0.137	0.980 ± 0.131	0.960 ± 0.142

*sRANKL* Soluble receptor activator of nuclear factor – κB ligand, *PINP* Total procollagen type 1 amino-terminal propeptide

*^,^**^,^******p < 0.001, ***p = 0.048, ****p = 0.007 and *****p = 0.049 between middle-aged and older men

OPG significantly correlated with age, cystatin C, E2 and FTI (Table 2).

**Table 2:** Spearman’s correlation analysis between serum osteoprotegerin and the studied variables

	All men (*n* = 194)		Middle-aged men (≤59 years of age, *n* = 98)		Older men (>59 years of age, *n* = 96)	
	*r*	*p*	*r*	*p*	*r*	*p*
Age	0.359	<0.001	0.165	0.105	0.050	0.631
Body mass index	0.031	0.672	0.057	0.580	−0.036	0.725
sRANKL	−0.034	0.713	0.019	0.885	−0.038	0.774
sRANKL/Osteoprotegerin ratio	−0.234	0.010	−0.204	0.116	−0.199	0.131
Total 17-β-Estradiol	0.160	0.028	0.164	0.110	0.211	0.043
Total testosterone	−0.025	0.729	0.106	0.302	−0.064	0.540
Sex hormone binding globulin	0.070	0.342	0.199	0.052	0.060	0.568
Free Estradiol Index	−0.072	0.323	−0.084	0.414	0.048	0.648
Free testosterone Index	−0.230	0.001	−0.142	0.167	−0.169	0.106
Parathyroid hormone	−0.076	0.300	−0.172	0.093	−0.009	0.930
25-hydroxy-vitamin D	−0.123	0.091	−0.115	0.085	−0.030	0.774
Osteocalcin	−0.102	0.161	−0.037	0.717	−0.040	0.706
C-terminal telopeptides of type-I collagen	−0.100	0.171	−0.095	0.358	−0.053	0.611
PINP	−0.043	0.553	−0.006	0.951	−0.044	0.667
L1-L4 bone mineral density	0.026	0.716	−0.041	0.689	0.010	0.923
Femur neck bone mineral density	−0.074	0.307	−0.071	0.485	−0.001	0.995
Cystatin C	0.298	<0.001	0.022	0.832	0.322	0.002
Creatinine	0.038	0.618	−0.085	0.415	0.132	0.239
MDRD-eGFR	−0.071	0.351	0.083	0.428	−0.142	0.207
Cystatin C-eGFR	−0.343	<0.001	−0.031	0.766	−0.335	0.001
Cystatin C and creatinine-eGFR	−0.231	0.003	0.023	0.834	−0.262	0.020

*sRANKL* Soluble receptor activator of nuclear factor – κB ligand, *PINP* Total procollagen type 1 amino-terminal propeptide

Furthermore, Cystatin C correlated with age (Spearman’s rho = 0.377, *p* < 0.001), creatinine (Spearman’s rho = 0.487, *p* < 0.001), MDRD eGFR (Spearman’s rho = −0.524, *p* < 0.001), E2 (Spearman’s rho = 0.222, *p* = 0.003), P1NP (Spearman’s rho = 0.151, *p* = 0.040) and OPG (Spearman’s rho = 0.298, *p* < 0.001). Although OPG did not correlate with serum creatinine or MDRD eGFR, it did show a significant correlation with cystatin C-eGFR, and cystatin C and creatinine-eGFR.

Multivariate linear regression analysis was carried out to determine the statistically significant predictors of serum OPG levels. Including age, cystatin C, FTI and E2 in a regression model showed that age, cystatin C, FTI and E2 were significant predictors of serum osteoprotegerin levels (Table 3).

**Table 3:** Predictors of osteoprotegerin using multiple regression analysis

	Osteoprotegerin	
	ß	*p*
Age	0.232	0.002
Total 17-β-Estradiol	0.166	0.021
Free testosterone Index	−0.178	0.012
Cystatin C	0.182	0.015

In order to reduce the confounding effect of age, using the median age of 59 years, the study population was divided into a middle-aged (those ≤ 59 years of age, *n* = 98) and an older (those > 59 years of age, *n* = 96) sub-group. Compared to the middle-aged (age: ≤ 59 years, *n* = 98), older men had significantly higher serum OPG and significantly lower RANKL levels and RANKL/OPG ratios (Table 1). The middle-aged individuals showed no significant correlation between serum OPG and sRANKL levels and the other studied parameters (Table 2). The older men showed a significant correlation between serum OPG levels and cystatin C. Including cystatin C and E2 in a regression model showed that cystatin C (standard regression coefficient (β) = 0.345; *p* = 0.002) was the only significant predictor of serum OPG levels in the older men.

There was no statistically significant difference in the group with non-detectable (<0.02 pmol/L, *n* =74) sRANKL concentrations as compared to those with detectable (≥0.02 pmol/L, *n* =120) sRANKL concentrations in the studied parameters, except for LS (1.190 (0.810-1.892) vs. 1.143 (0.749-1.855) gm/cm^2^; *p* = 0. 043) and FN (0.984 (0.646-1.281) vs. 0.949 (0.602-1.331) gm/cm^2^; *p* = 0.049) BMD. As such, LS and FN BMD is significantly lower in those with undetectable sRANKL levels.

There was no difference in OPG, sRANKL and sRANKL/OPG ratio upon comparing the vitamin D sufficient (25-OH-D ≥ 75 nmol/L) with the vitamin D insufficient (25-OH-D < 75 nmol/L) individuals.

## Discussion

Our results demonstrate that serum OPG levels increase and sRANKL/OPG ratio decreases with age in healthy men over 50 years of age. With regards to the relationship of OPG with age, our findings are in tune with those reported by others (7–10,12,14-19). As enumerated in Table 4, among the various predictors studied, perhaps the positive correlation of OPG with age is the only finding showing consensus in the different studies to date. The only exception with this regards is the non-significant finding by Oh et al., where the lack of correlation may be due to the limited number of cases studied (*n* = 80) and a relatively lower age maximum of the study population (i.e., 70 years).

**Table 4:** Literature data on correlation coefficients (with p values) between serum osteoprotegerin levels in men and the studied parameters

Authors	Age	BMD	T	E2	FEI	FTI	PTH	25-OH-D	Creatinine	Bone markers
Szulc et al. [7]	*r* = 0.41	n.s.	n.s.	n.s.	^a^ *r* = 0.18	^a^ *r* = 0.31	*r* = −0.23	n.s.	n.s.	^b^ *r* = −0.20 *p* < 0.01
	*p* = 0.0001				*p* < 0.02	*p* = 0.0001	*p* < 0.01			
Khosla et al. [8]	*r* = 0.39	^c^ *r* = −0.17	*r* = −0.16	n.s.	^d^n.s.	^e^ *r* = −0.27	-	-	-	^f^ *r* = 0.16 *p* < 0.05
	*p* < 0.001	*p* < 0.05	*p* < 0.05			*p* < 0.001				^g^ *r* = 0.26 *p* < 0.001
Khosla et al. [9]	-	-	-	-	-	-	-	-	-	^h^n.s.
Kudlacek et al. [10]	^i^ *p* < 0.05	n.s.	*r* = 0.1	-	-	-	*r* = −0.17 *p* < 0.0001	n.s.	-	-
			*p* < 0.05							
Trofimov et al. [12]	*r* = 0.42	n.s.	n.s.	n.s.	-	-	-	-	-	-
	*p* < 0.001									
Oh et al. [13]	n.s.	^j^ *r* = −0.259	n.s.	*r* = −0.319	-	-	-	-	-	^k^ *r* = −0.25 *p* = 0.024
		*p* = 0.020		*p* =0.004						
Indridason et al. [14]	^i^ *p* < 0.05	^l^ *r* = −0.13 p ≤ 0.05	-	-	^n^ *r* = 0.15	^o^ *r* = 0.09 p ≤ 0.001	n.s.	n.s.	^p^-	^q^ *r* = −0.09 p ≤ 0.05
		^m^ *r* = −0.11 p ≤ 0.05			p ≤ 0.05					
Mazziotti et al. [15]	*r* = 0.37 *p* = 0.002	-	-	-	-	-	-	-	^r^ *r* = 0.46	^s^ *r* = 0.47 p ≤ 0.05
									p ≤ 0.05	
Stern et al. [16]	*r* = 0.552	^t^ *p* < 0.05	-	-	-	-	-	-	-	-
	*p* < 0.001									
Samelson et al. [17]	^u^ *p* < 0.05	^v^n.s.	-	-	-	-	-	-	^w^ *p* < 0.05	-
Jorgensen et al. [18]	*r* = 0.52	^x^ *p* = 0.001	-	-	-	-	-	-	-	-
	*p* < 0.001									
Szulc et al. [19]	-	^y^ *r* = −0.28	-	-	-	-	-	-	-	^z^ *r* = −0.10 *p* < 0.05
		*p* < 0.001								^aa^ *r* = 0.25 *p* < 0.001
										^ab^ *r* = 0.14 *p* < 0.05
This study	*r* = 0.359 *p* < 0.001	n.s.	n.s.	*r* = 0.16 *p* = 0.028	n.s.	*r* = −0.23 *p* = 0.001	n.s.	n.s.	^ac^n.s.	n.s.

*BMD* bone mineral density, *T* testosterone, *E2* 17β-estradiol, *FEI* free estradiol index, *FTI* free testosterone index, *PTH* parathyroid hormone, *25-OH-D* 25-hydroxyvitamin D, *n.s.* statistically non significant

^a^After adjustment for age and body weight in > 40 years of age, ^b^urinary total deoxypyridinoline (DPD) after adjustment for age in > 40 years of age, ^c^mid-radius in ≥ 50 years of age, ^d^ *r* = −0.18 (*p* < 0.05) for bioavailable E2 in ≥ 50 years of age, ^e^ *r* = −0.27 (*p* < 0.001) for bioavailable T in ≥ 50 years of age, ^f^urinary N-telopeptide of type I collagen (NTx), ^g^urinary free DPD, ^h^urinary NTx and DPD, ^i^statistically significant positive correlation, but r value is not mentioned, ^j^lumbal spine (LS), ^k^osteocalcin (OC), ^l^total body, ^m^hip, ^n,o^calulated free sex hormone levels, ^p^n.s. for cystatin C, ^q^serum C-terminal cross-linked telopeptides of type I collagen (CTx), ^r^in a group including men and women (*n* = 52) aged between 65 and 84 years, ^s^serum CTx in a group including men and women (*n* = 52) aged between 65 and 84 years, ^t^LS (statistically significant positive correlation, but r value is not mentioned), ^u^age increased (Trend, *p* < 0.05) with increasing quartile of osteoprotegerin (OPG), ^v^age-adjusted femur neck BMD showed no difference between OPG quartiles, ^w^glomerular filtration rate (GFR) decreased (Trend, *p* < 0.05) with increasing quartile of OPG, ^x^age-adjusted distal forearm BMD decreased (Trend, *p* = 0.001) with increasing OPG tertile, ^y^total (distal radius and tibia) ^v^BMD above the median OPG level, ^z^OC and N-terminal extension type 1 collagen propeptide (P1NP) below the median OPG level, ^aa^urinary DPD and ^ab^serum CTx above the median OPG level, ^ac^n.s. with creatinine and GFR but *r* = 0.298 (*p* < 0.001) with cystatin C

With regards to the correlation of OPG with BMD and biochemical markers of bone turnover, as per the results of the studies published to date, statistically significant positive, negative and non-significant findings have been presented (see Table 4). Our finding of non-significance only contributes to the need for further studies. Knowing that OPG messenger ribonucleic acid (mRNA) is expressed in a variety of tissues, including lung, kidney and heart, multiple tissues contribute together to circulating OPG, as such measurement of OPG levels in the bone microenvironment is most desirable [27].

Our finding of statistically significant positive correlation between E2 with OPG, along with those reported by Schulz et al. and Indridason et al., supports the finding that E2 increases OPG mRNA steady state levels and protein production in a human estrogen-responsive osteoblastic cell line [4]. Furthermore, Khosla et al. have demonstrated that estrogen treatment increases OPG levels in adult men [9]. Our finding of negative correlation between testosterone and OPG supports two and disagrees with another 2 previous studies [5, 7, 8, 14]. Nonetheless, the negative correlation supports the finding by Khosla et al. where it was demonstrated in vivo that testosterone therapy resulted in lower OPG levels [9].

Our finding of non-correlation of age with sRANKL is in accordance with that reported earlier [11–13, 15, 16, 18]. Upon bivariate analysis we found no correlation between sRANKL and BMD, this finding is in agreement with that of Trofimov et al. and Oh et al., but in disagreement with the findings of Stern et al., who reported an inverse association between RANKL and BMD [12, 13, 16]. Nonetheless, we observed statistically higher FN and LS BMD in those with detectable (higher) sRANKL levels, this finding, at least in part, may explain the low risk of non-traumatic fracture in participants in the highest tertile of RANKL in the study by Schett et al. [11]. It needs to be pointed out that Schett et al. found no relationship between sRANKL and bone ultrasound data and they did not carry out BMD measurements in their study population [11]. Adding to the controversy is the finding by Jorgensen et al., where they found no difference in BMD between those with detectable versus non-detectable sRANKL levels [18]. Although the present generation of sRANKL immunoassays has a better lower detection limit than its predecessors, methodology with improved detection limits are most desired. Probably introduction of more sensitive immunoassays may help quantify undetectable sRANKL levels and perhaps may help explore relationships between those with low sRANKL and its known predictors.

As far, the one study that studied the relationship of sRANKL/OPG ratio with age found no significance with this regards, in contrast to this our study reports a negative correlation between the ratio and age. This finding certainly needs validation by others.

Although it is suggested that PTH has a suppressive effect on OPG production, the present study did not find any correlation between PTH and OPG [28, 29]. Our results are in agreement with those of Indridason et al. and in contrast to those of Szulc et al. and Kudlacek et al. [7, 10, 14].

In contrast to the finding that 1,25 dihydroxyvitamin D stimulates OPG production, in vivo studies in healthy men, including the present study, have not found any correlation between 25-OH-D, an index of body vitamin D status, and OPG [7, 10, 14, 30, 31].

In summary, the non-agreement between findings from different studies may be explained, at least partially, by the different assay methodology, in some cases the use of frozen samples more than a decade old, the different recruitment criteria used in the selection of the cohort, the size of the study sample and perhaps the age composition of the cohorts studied.

The role of decreased protein clearance as a potential cause of age-related OPG elevation has been considered in a few previous studies [15, 17, 19]. Mazzioti et al. found a significant positive correlation between creatinine and OPG, and Samelson et al. found that with increasing quartiles of OPG the GFR decreased significantly [15, 17]. This finding is in contrast to the results of Szulc et al., where no correlation was found between OPG and creatinine [19]. Our findings show that there is no correlation between OPG and eGFR, when the MDRD equation is used, but there is a significant negative correlation using the CKD-EPI cystatin C and the CKD creatinine-cystatin C equations. This finding may illustrate the importance of the type of equation used to calculate eGFR. Nonetheless, the combined creatinine-cystatin C equation is considered to perform better than equations based on either of these markers alone [25, 26]. We found a strong positive correlation between OPG and cystatin C. Our positive finding is in contrast to that of Indridason et al., where no such correlation was found [14]. Although the assay methodology used by Indridason et al. is not the one used by us, this difference alone does not explain the difference in the observations of the two studies. Nonetheless, serum OPG has been shown to correlate positively in male patients with chronic renal failure [32].

Although the study participants were members of a well-defined healthy cohort randomly selected from the population, there are limitations to our study. The HunMen study was not designed primarily for the aim of this manuscript, and only 194 samples were available for serum OPG and sRANKL determination from a total of 206 participants [20]. In addition, we were not in a position to study the effects of bioavailable (free) sex hormones on OPG, instead we used calculated free sex hormone indices to carry out the different statistical analysis. Although the mechanism of the effect, as a renal marker or inhibitor of osteoclastogenesis, of cystatin C on OPG levels cannot be elucidated by the findings of the present study, we have found that it is a significant predictor of serum OPG levels. These findings need verification and further studies are necessary.

The present study confirms to the STROBE Statement, a checklist of items that should be included in reports of observational studies has been provided as Additional file 1 [33].

## Conclusion

The present study demonstrated that in addition to age, the stronger predictor, other adjustable factors such as cystatin C, FTI and E2 were also significant predictors of OPG. Furthermore, the association between cystatin C and OPG was more evident with increased age. In conclusion, cystatin C is a significant predictor of OPG independently of age, FTI and E2.

## Additional file

Additional file 1:
**STROBE Statement—checklist of items that should be included in reports of observational studies.**
